# Prediction of the risk of severe small bowel obstruction and effects of Houpu Paiqi mixture in patients undergoing surgery for small bowel obstruction

**DOI:** 10.1186/s12893-024-02343-0

**Published:** 2024-02-17

**Authors:** Ze-zheng Wang, Zhe-kui Liu, Wen-xing Ma, Yun-hua Wu, Xiang-long Duan

**Affiliations:** 1 The Second Department of General Surgery, Shaanxi Provincial People’s Hospital, 256 West Youyi Road, Xi’an, Shaanxi 710068 China; 2 Yan’an University, Yan’an, 716000 China

**Keywords:** Severe small bowel obstruction, Risk factors, Houpu Paiqi mixture, Prediction model, Nomogram

## Abstract

**Aim:**

Small bowel obstruction is a common condition that requires emergency surgery. Slow recovery of bowel function after surgery or the occurrence of one or more complications can exacerbate the disease and result in severe small bowel obstruction (SSBO), significantly impacting recovery. It is characterized by a failure to regain enteral nutrition promptly, requiring long-term intensive care. Therefore, it is necessary to identify factors that predict SSBO, to allow early intervention for patients likely to develop this condition.

**Methods:**

Of the 260 patients who underwent emergency or elective surgery for small bowel obstruction between January 2018 and December 2022, 45 developed SSBO. The least absolute shrinkage and selection operator regression model was applied to optimize factor selection and multivariable logistic regression analysis was used to construct a predictive model. The performance and clinical utility of the nomogram were determined and internal validation was conducted. In addition, the effects of the Houpu Paiqi mixture on postoperative recovery were analyzed by comparing the clinical data of 28 patients who were treated with the mixture and 61patients who did not receive it.

**Results:**

The predictors included in the prediction nomogram were age, peritonitis, intestinal resection and anastomosis, complications, operation time, Acute Physiology and Chronic Health Evaluation II score, white blood cell count, and procalcitonin level. The model had an area under the receiver operating characteristic curve of 0.948 (95% confidence interval: 0.814–0.956). Decision curve analysis demonstrated that the SSBO risk nomogram had a good net clinical benefit. In addition, treatment with the Houpu Paiqi mixture reduced postoperative exhaust time, postoperative defecation time, time to first postoperative liquid feed, and length of stay in hospital.

**Conclusions:**

We developed a nomogram that can assist clinicians in identifying patients at greater risk of SSBO, which may aid in early diagnosis and intervention. Additionally, we found that the Houpu Paiqi mixture promoted postoperative recovery.

## Introduction

Small bowel obstruction is a common condition that requires urgent surgical treatment [1, 2]. Despite advancements in surgical techniques [3–5], efficacy is limited by various factors, including a high postoperative complication rate and slow intestinal function recovery. In certain instances, small bowel obstruction can progress to severe small bowel obstruction (SSBO), which refers to a type of small bowel obstruction that is challenging to recover from in terms of postoperative intestinal function. It is characterized by a failure to regain enteral nutrition promptly and is often complicated by one or more additional issues such as pulmonary infections, severe hypoproteinemia, septic shock, and even death, requiring long-term intensive care. As well as the impact on patient health, SSBO prolongs hospital stays, increases costs, and reduces patient confidence and compliance [6]. Identifying the critical factors affecting postoperative recovery in patients with intestinal obstruction, particularly those who are critically ill, may allow early intervention, thus improving outcomes. However, few studies have been conducted on the identification and postoperative recovery of patients with SSBO.

Several factors influence the postoperative recovery of patients with small bowel obstruction, one of most crucial being the speed of postoperative intestinal function recovery. Prolonged intestinal obstruction leads to intestinal wall edema and local inflammation, making recovery after surgery more challenging and aggravating obstructive symptoms such as abdominal pain and distension. In addition, persistent local inflammation increases the likelihood of systemic infections. Traditional Chinese medicine has long been recognized for its ability to aid in the recovery of intestinal function [7], with Houpu Paiqi mixture being widely utilized and resulting in positive outcomes. Previous studies have demonstrated that the key constituents of Houpu Paiqi mixture possess anti-inflammatory properties, reduce oxidative stress, enhance intestinal peristalsis, and minimize postoperative adhesion formation [8–11].

This study aimed to identify potential risk factors associated with SSBO, establishing an independent prediction model for this complication. This would provide clinicians with a reliable tool for identifying patients at risk of developing SSBO after surgery. We also evaluated the clinical effectiveness of Houpu Paiqi mixture in the postoperative treatment of patients with small bowel obstruction.

## Methods and materials

### Study design and patients

**Fig. 1:**
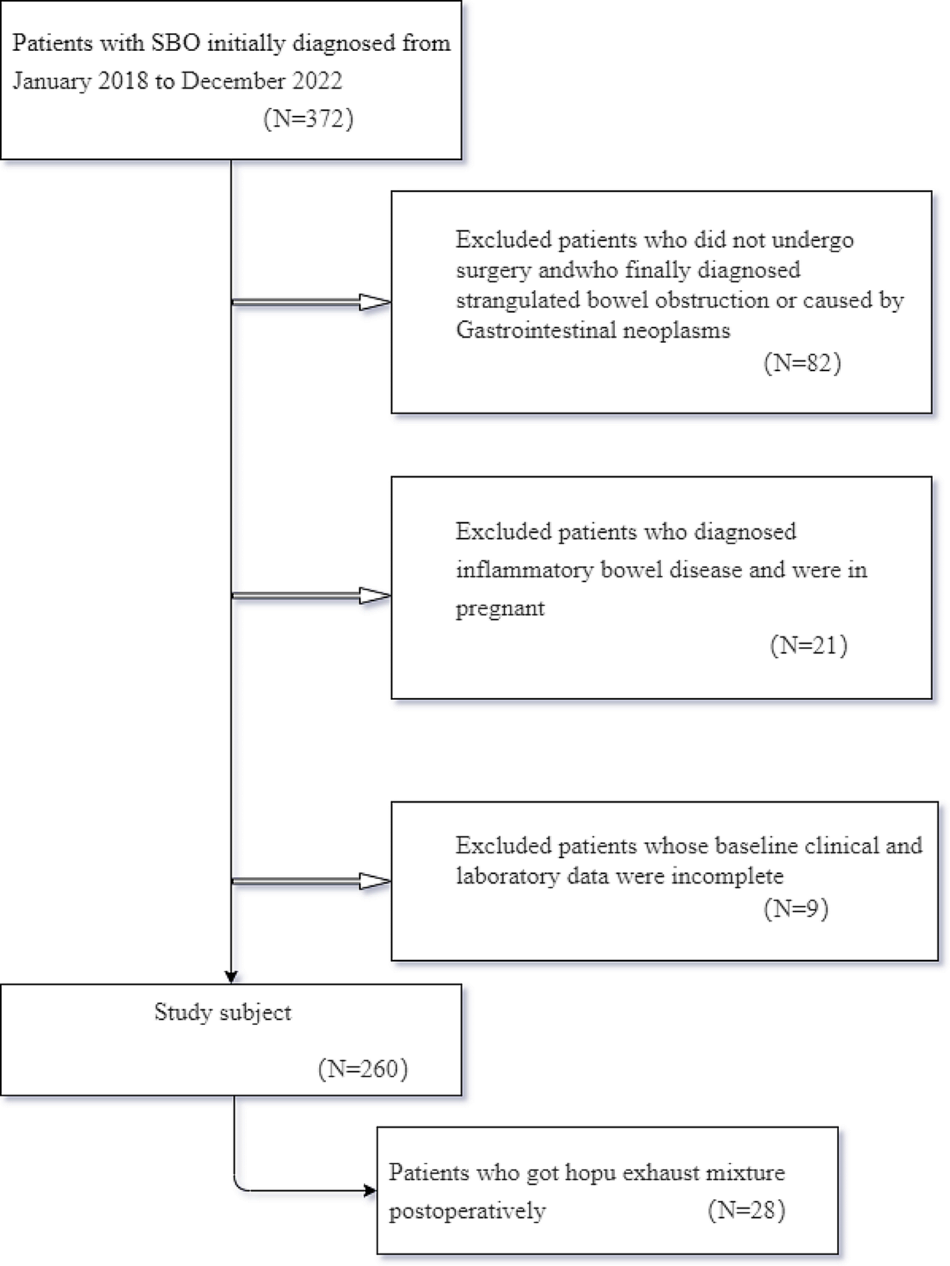
Flowchart of exclusion criteria for patient inclusion

This retrospective observational study collected and reviewed clinical data from 372 patients with small bowel obstruction who were admitted to Shaanxi Provincial People’s Hospital between January 2018 and December 2022; 28 of these patients were treated with Houpu Paiqi mixture after surgery. The exclusion criteria were as follows: (1) did not undergo surgery or mechanical strangulation obstruction; (2) gastrointestinal or abdominal tumors; (3) inflammatory bowel disease or pregnancy; (4) poor general health, including unconsciousness or severe cardiopulmonary insufficiency, which would affect the observation indicators; and (5) incomplete clinical data. Finally, 260 patients were included in this study. (Fig. 1). A small amount of liquid diet is initially provided post-flatus and defecation, before gradually transitioning to a full liquid diet and eventually a normal diet. The study was approved by Ethics Committee of Shaanxi Provincial People’s Hospital and Informed consent was obtained from all patients.

### Data collection

Information on baseline demographic characteristics, including age and sex, were obtained from patients’ electronic medical records downloaded from the hospital information system. Clinical parameters such as body mass index (BMI), attending physician, presence of peritonitis, presence of chronic diseases (hypertension and diabetes), history of abdominal surgery, type of surgery, duration of surgery, intraoperative blood loss, postoperative complications, imaging data, laboratory indicators, and Acute Physiology and Chronic Health Evaluation (APACHE) II [12, 13] and Nutritional Risk Screening 2002 [14, 15] scores were also analyzed. Laboratory indicators included baseline and postoperative white blood cell (WBC) counts, red blood cell counts, hemoglobin levels, albumin levels, creatinine levels, procalcitonin levels, and blood gas analysis. Imaging was performed within 24 h of admission or postoperatively [16], and findings included multiple air and fluid levels on standing plain abdominal radiography, severe obstruction on abdominal computed tomography (CT), adhesion formation on abdominal CT, small intestinal fecal sign on abdominal CT, and abdominal fluid accumulation on abdominal CT. All patients underwent 64-slice spiral CT [17]. Many patients are unable to complete a CT examination after surgery. Therefore, it is necessary to refer to the admission CT scan. Adhesive bands were defined as connections between the small intestine and the anterior abdominal wall or between segments of the small intestine. Severe obstruction was defined as the presence of continuous bowel thickening accompanied by clear signs of filling and collapse, and an obstruction with a minimum diameter at least three times smaller than its maximum diameter. Small bowel fecal sign was characterized by the presence of gas and fecal-like substances in the dilated intestinal cavity at the proximal end of the small intestinal obstruction. The APACHE II score was used to assess disease severity, with a higher APACHE II score indicating a greater risk coefficient and mortality rate. The scores were assessed at admission by two experienced clinicians, and the records were reviewed for accuracy.

Clinical data from 28 patients who received postoperative treatment with the Houpu Paiqi mixture were compared with data from 61 selected patients who did not receive the Houpu Paiqi mixture treatment. Both groups were hospitalized from January 2022 to December 2022. After 2022, standardized treatment protocols for patients with small bowel obstruction were implemented within the General Surgery department. These protocols include specific indications, usage, and dosage of the Houpu Paiqi mixture. As a result, the data from after 2022 shows a certain degree of homogeneity and is considered for inclusion in the Houpu Paiqi study. Nevertheless, this did not affect the diagnosis and treatment of small bowel obstruction and SSBO.

For the patients who were treated by Houpu Paiqi mixture received an enema each day after surgery, Houpu Paiqi mixture (Ruiyang Pharmaceutical Co., Ltd, Shandong, China) 50mLwas dissolved into 250mL saline.

In addition to the baseline demographic and clinical characteristics listed above, data on the length of hospital stay, postoperative exhaust time, time to first postoperative defecation, time to first postoperative liquid feed, drainage tube withdrawal time, nasogastric tube withdrawal time, and WBC counts after treatment were collected.

### Statistical analysis

Patients who developed SSBO and those who did not were compared using one-way analysis of variance, the Kruskal–Wallis test, the Mann–Whitney U test, and Pearson’s chi-squared test, as appropriate. Patients who were treated with the Houpu Paiqi mixture and those who were not were compared in the same way. Data are expressed as the mean ± standard deviation or median (quartile) for continuous variables, and frequency and percentage for categorical variables, based on the normal distribution of the data.

To identify potential predictive factors for SSBO, we employed the least absolute shrinkage and selection operator (LASSO) regression, which is an effective prediction method in high-dimensional datasets [18, 19]. The optimal value of λ was determined using ten-fold cross-validation. Subsequently, a multivariable logistic regression analysis was performed using the factors identified by the LASSO regression model. Significant predictors were selected using the backward elimination (Wald) method and used to construct a nomogram. The predictive factors were reported as a forest plot with regression coefficient (β), odds ratios (ORs), 95% confidence intervals (CIs), and *P*-values. Statistical significance was set at *P* < 0.05.

A calibration curve was plotted to evaluate the discriminatory ability of the nomogram. Decision curve analysis (DCA) was performed to assess the clinical application of the SSBO nomogram by measuring the net benefits at various threshold probabilities [20]. Internal validation was performed by bootstrapping with 500 replicates.

## Results

### Baseline patient characteristics and risk factors of SSBO

**Fig. 2:**
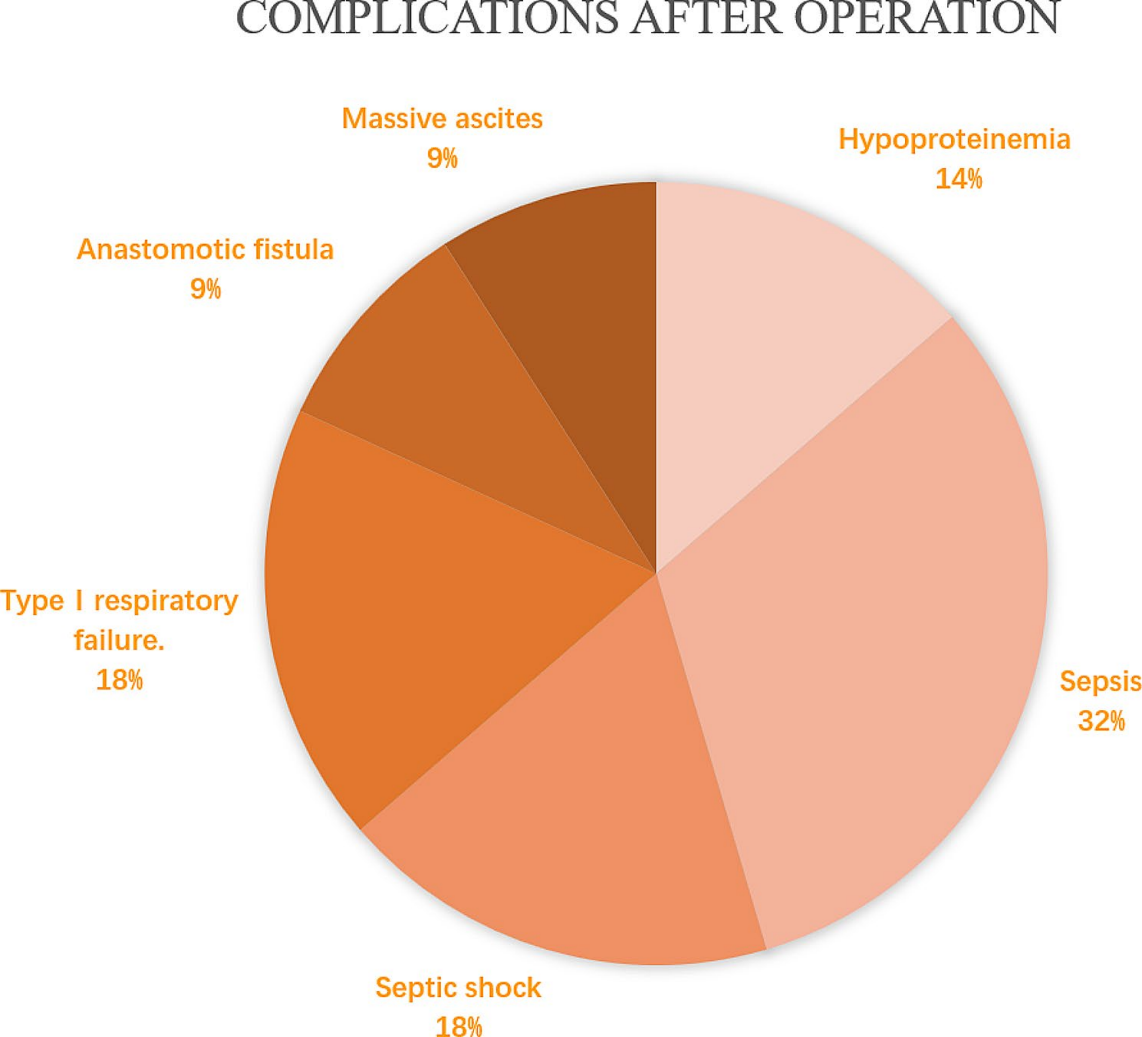
Complications after surgery

**Fig. 3:**
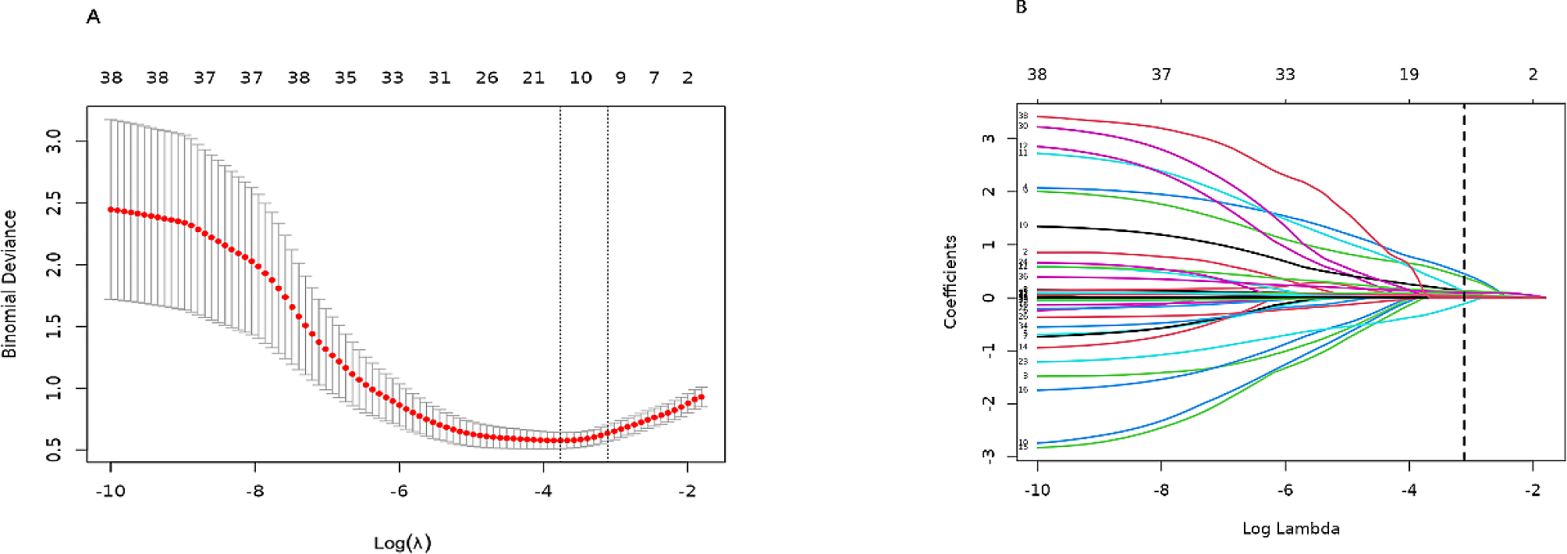
Features selection using the LASSO binary logistic regression model. **A** Optimal parameter (lambda) selection in the LASSO model using ten‑fold cross‑validation through the minimum criteria. Dotted vertical lines were drawn at the optimal values using the minimum criteria and 1 SE of the minimum criteria (the 1‑SE criteria). **B** LASSO coefficient profiles of the 37 factors. A coefficient profile plot was generated against the log(lambda) sequence. A vertical line was drawn at the value selected using ten‑fold cross‑validation, where optimal lambda resulted in 8 features with non-zero coefficients. LASSO, least absolute shrinkage and selection operator; SE, standard error

**Fig. 4:**
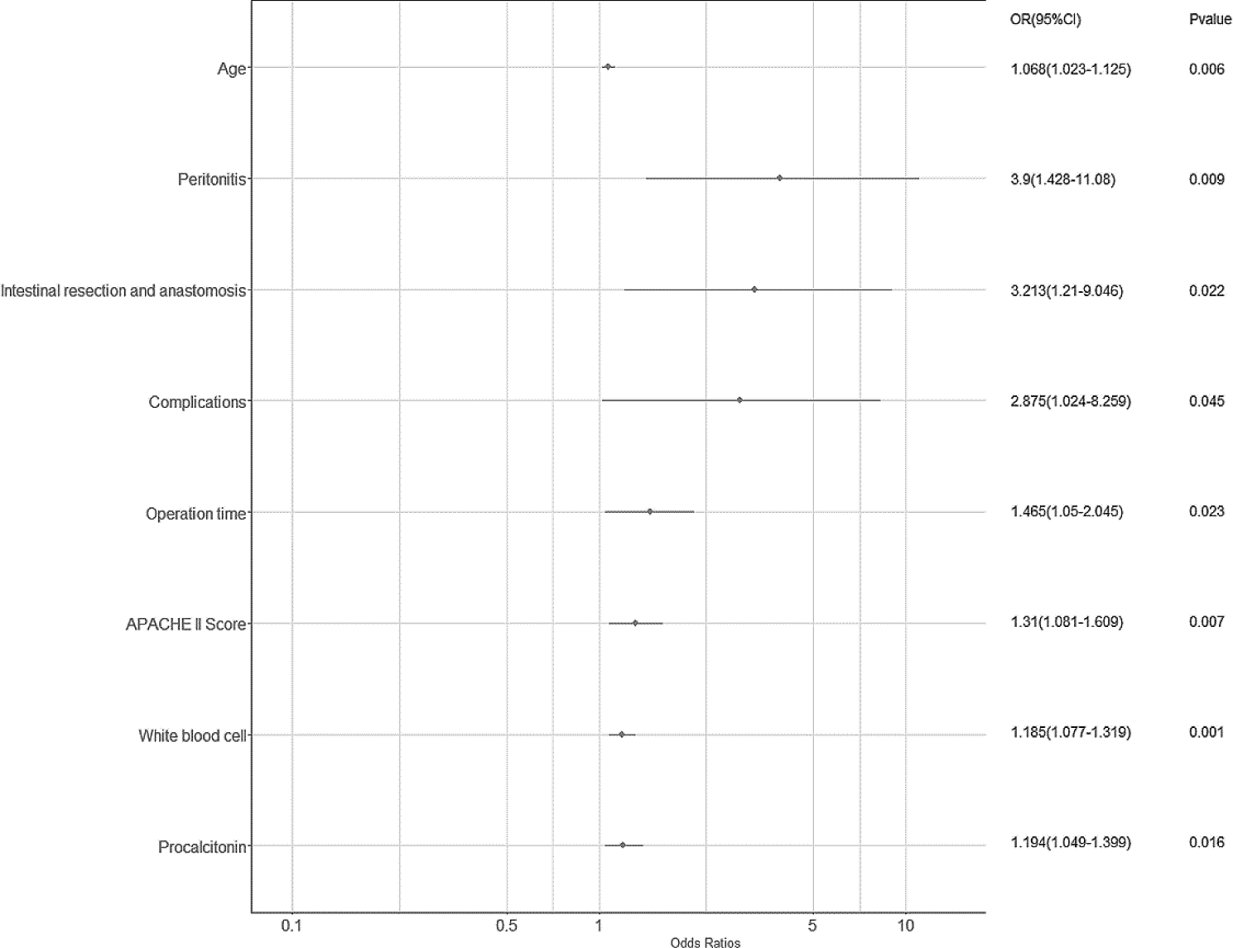
ORs were estimated using a logistic regression model and the backward elimination (Wald) method. The regression coefficient was denoted as β. CI, confidence interval; OR, odds ratio

Of the 260 patients included in this study, 45 (17.3%) had SSBO, and 30 of these patients developed single-organ or systemic complications, with sepsis and septic shock being the most common (Fig. 2). Table 1 presents the baseline demographic information, clinical parameters, and laboratory results of patients with and without SSBO. Of the 37 variables, 8 were selected based on non-zero coefficients calculated using LASSO (logistic regression analysis Fig. 3). The selected factors were: age, peritonitis, intestinal resection and anastomosis, complications, operation time, APACHE II score, WBC count, and postoperative complications. Subsequently, these features were included in a multivariable logistic regression analysis and confirmed to be independent risk factors for SSBO (Table 2; Fig. 4).

**Table 1:** Patient characteristics

Characteristics	Total (*n*=260)	No SSBO(*n*=215)	SSBO (*n*=45)	*P*-value
Age(years)	61.0(50; 68)	59(46; 66)	70(62; 75)	<0.001^*^
Gender				0.937
Male Female	166(63.8%) 94(36.2%)	138(64.2%) 77(35.8%)	28(62.2%) 17(37.8%)	
Doctor in charge				0.202
Senior seniority Low seniority	206(79.2%) 54(20.8%)	174(80.9%) 41(19.1%)	32(71.1%) 13(28.9%)	
Peritonitis:				<0.001^*^
Yes No	61(23.5%) 199(76.5%)	38(17.7%) 177(82.3%)	23(51.1%) 22(48.9%)	
Abdominal pain				0.664
Yes No	200(76.9%) 60(23.1%)	167(77.7%) 48(22.3%)	33(73.3%) 12(26.7%)	
Abdominal distention				0.745
Yes No	243(93.5%) 17(6.5%)	200(93.0%) 15(6.98%)	43(95.6%) 2(4.44%)	
Vomiting				0.193
Yes No	196(75.4%) 64(24.6%)	166(77.2%) 49(22.8%)	30(66.7%) 15(33.3%)	
Extensive adhesion and separation				0.003
Yes No	165(63.5%) 106(40.8%)	118(54.9%) 97(45.1%)	36(80.0%) 9(20.0%)	
Intestinal resection and anastomosis				<0.001^*^
Yes No	113(43.5%) 147(56.5%)	79(36.7%) 136(63.3%)	34(75.6%) 11(24.4%)	
Chronic diseases				0.484
Yes No	165(63.5%) 95(36.5%)	139(64.7%) 76(35.3%)	26(57.8%) 19(42.2%)	
Complications				0.005
Yes No	64(24.6%) 196(75.4%)	45(20.9%) 170(79.1%)	19(42.2%) 26(57.8%)	
Presence of gas and liquid in X-ray				0.994
Yes No	211(81.2%) 49(18.8%)	175(81.4%) 40(18.6%)	36(80.0%) 9(20.0%)	
Severe obstruction in CT				0.748
Yes No	165(63.5%) 95(36.5%)	135(62.8%) 80(37.2%)	30(66.7%) 15(33.3%)	
Adhesive band in CT				0.062
Yes No	126(48.5%) 134(51.5%)	98(45.6%) 117(54.4%)	28(62.2%) 17(37.8%)	
Small intestinal fecal sign in CT				0.373
Yes No	81(31.2%) 179(68.8%)	70(32.6%) 145(67.4%)	11(24.4%) 34(75.6%)	
Ascites in CT				0.710
Yes No	125(48.1%) 135(51.9%)	105(48.8%) 110(51.2%)	20(44.4%) 25(55.6%)	
Abdominal operations times				0.173
0 1 2 3	94(36.2%) 113(43.5%) 47(18.1%) 6(2.31%)	81(37.7%) 87(40.5%) 42(19.5%) 5(2.33%)	13(28.9%) 26(57.8%) 5(11.1%) 1(2.22%)	
Operation time(hour)	2.5(1.90; 3.5)	2.45(1.67; 3.4)	3.2(2.50; 4.50)	<0.001^*^
Blood loss(ml)	50.0(40.0; 95.0)	50.0(35.0; 95.0)	50.0(40.0; 85.0)	0.699
APACHE II Score	5.0(4.0; 8.0)	5.0(3.0; 7.0)	8.0(7.0; 11.0)	<0.001^*^
BMI	22.3(20.0; 24.0)	22.2(20.0; 24.0)	23.1(20.1; 23.9)	0.723
Body temperature(°C)	36.3(36.1; 36.6)	36.3(36.1; 36.7)	36.2(36.2; 36.4)	0.360
Heart rate	82.0(78.0; 96.0)	80.0(77.5; 98.0)	96(80.0; 96.0)	0.180
White blood cell(×10^9^/L)	6.67(5.29; 9.98)	6.42(5.16; 8.84)	10.6(6.71; 13.8)	<0.001^*^
Postoperative white blood cells	9.71(7.24; 12.5)	9.48(7.17; 12.2)	10.5(7.54; 16.5)	0.071
Hemoglobin(g/dL)	126(109; 140)	126(109; 140)	124(107; 135)	0.667
Serum creatinine(μmol /L)	72.0(57.7; 98.7)	73.0(59.1; 98.8)	68.5(45.8; 98.3)	0.330
Urea nitrogen(mmol/L)	5.45(3.91; 8.32)	5.20(3.75; 6.98)	8.67(5.19; 12.5)	<0.001^*^
Albumin(g/L)	32.9(±6.85)	33.3(±6.85)	30.9(±6.53)	0.026^*^
Potassium(mmol/L)	4.06(±0.57)	4.04(±0.56)	4.13(±0.59)	0.383
Sodium(mmol/L)	142(135; 144)	142(133; 145)	143(141; 143)	0.910
Procalcitonin	0.76(0.35; 1.76)	0.62(0.3; 1.34)	1.94(0.89; 7.33)	<0.001^*^
Oxygen partial pressure(mmHg)	89.0(73.0; 100)	92.0(73.0; 100)	82.0(69.0; 100)	0.384
Arterial blood PH	7.38(7.34; 7.47)	7.38(7.33; 7.47)	7.35(7.35; 7.49)	0.346

Categorical data are expressed as percentages, and continuous data are expressed as quartile; SSBO, severe small bowel obstruction; BMI, body mass index (kg/m2); APACHE, Acute Physiology and Chronic Health Evaluation

**Table 2:** Logistic regression analysis of factors predictive of severe small bowel obstruction

Characteristics	β	OR(95% CI)	*P*-value
Age	0.066	1.068(1.019-1.12)	0.006
Peritonitis	1.361	3.9(1.413-10.762)	0.009
Intestinal resection and anastomosis	1.167	3.213(1.186-8.704)	0.022
Complications	1.056	2.875(1.022-8.088)	0.045
Operation time	0.382	1.465(1.054-2.036)	0.023
APACHE II Score	0.27	1.31(1.076-1.596)	0.007
White blood cell	0.17	1.185(1.072-1.31)	0.001
Procalcitonin	0.178	1.194(1.033-1.381)	0.016

The regression coefficient was denoted as β. OR, odds ratio; CI, confidence interval

### Development of the risk prediction model

**Fig. 5:**
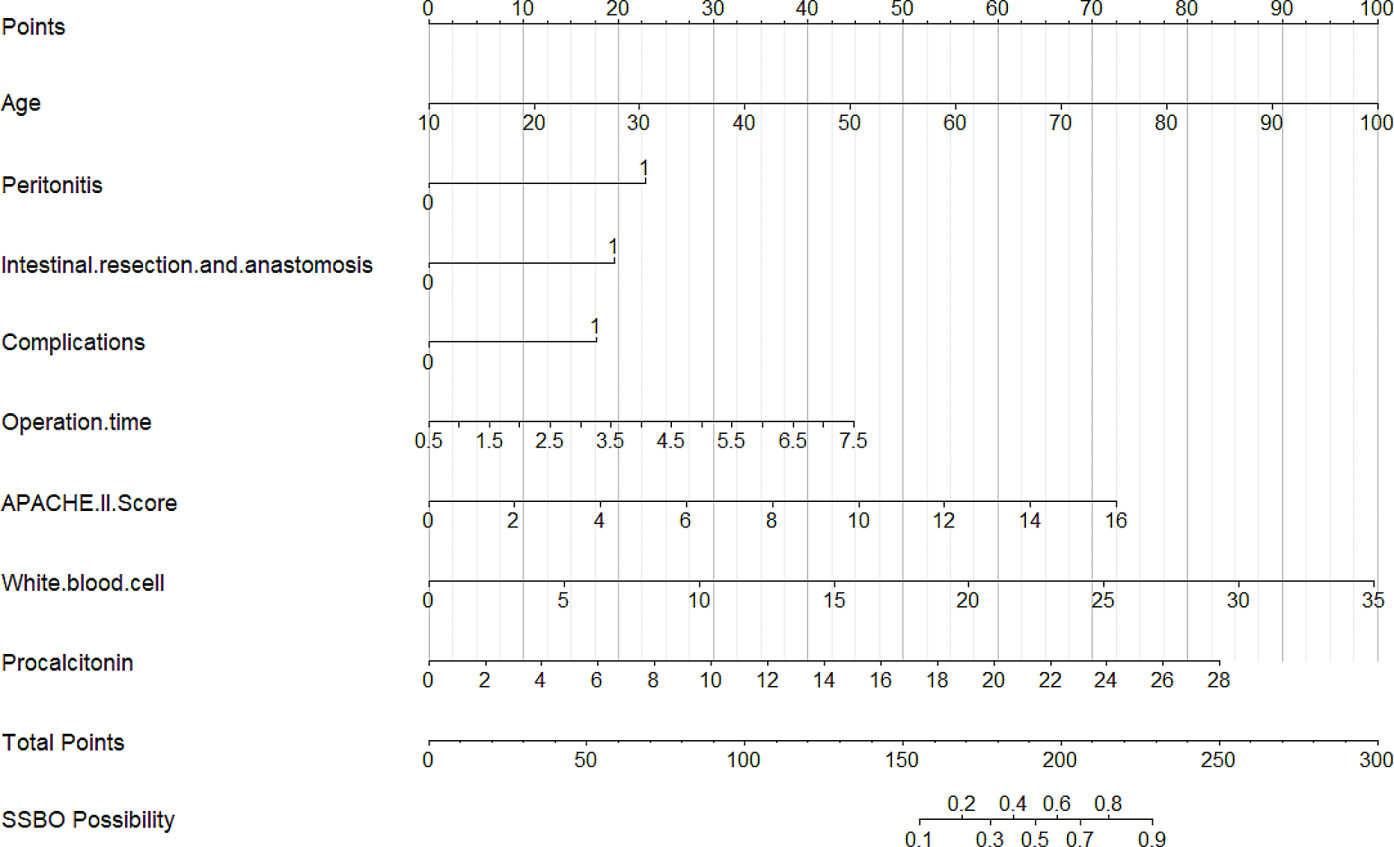
Nomogram for predicting SSBO risk. Each variable was assigned a score ranging from 0 to 100. The scores for each variable were added together, yielding a sum that can be located on the total points axis, predicting the probability of SSBO risk. SSBO, severe small bowel obstruction

Table 2 presents the results of the logistic regression analysis for age, peritonitis, intestinal resection and anastomosis, complications, operation time, APACHE II score, WBC count, and procalcitonin level. A nomogram (Fig. 5) was constructed to visualize the incorporation of these independent risk factors into a prediction model.

### Performance and validation of the SSBO risk nomogram

**Fig. 6:**
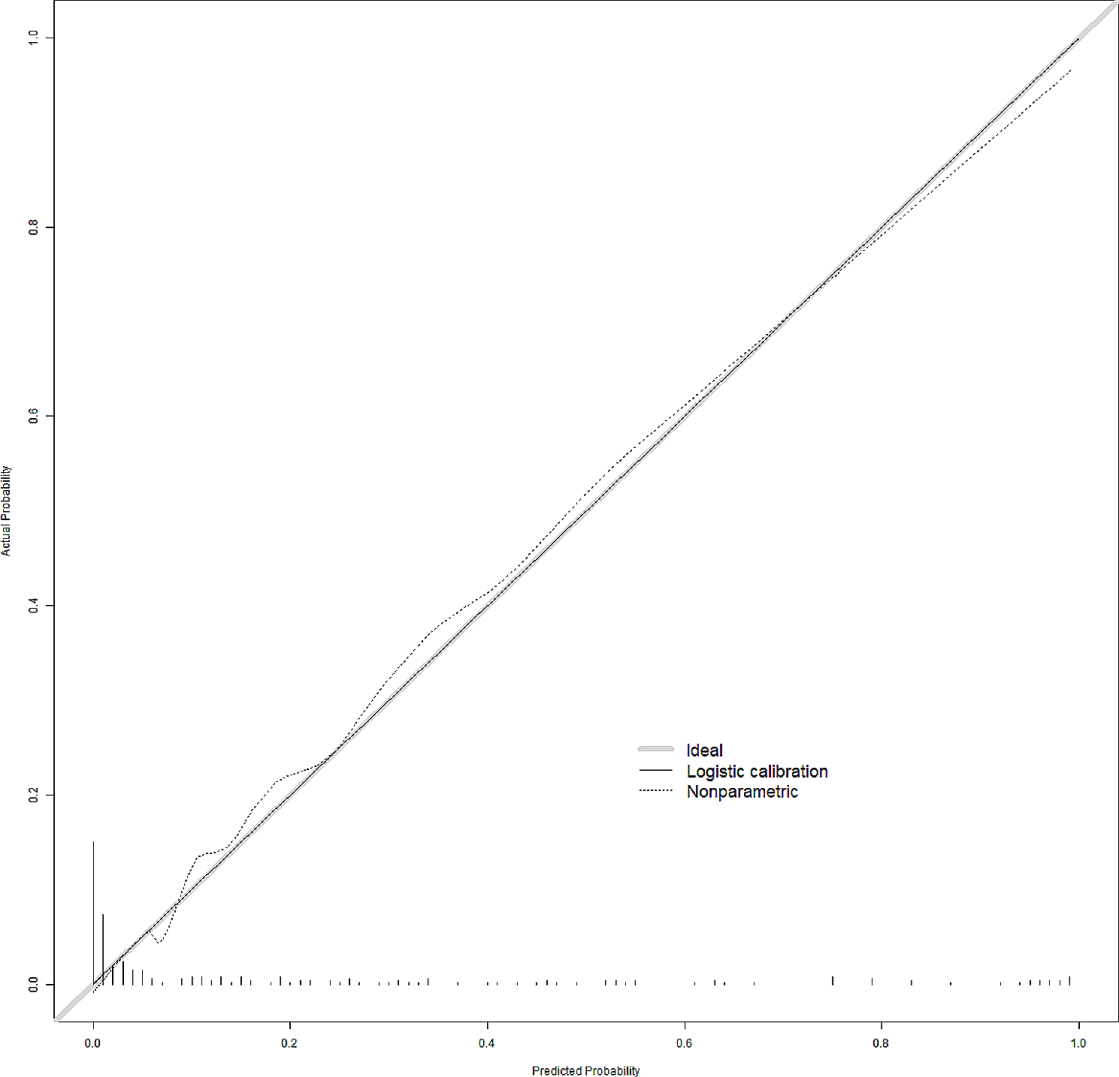
Nomogram calibration plot. As the solid line (performance nomogram) approached the dotted line (ideal model), the prediction accuracy of the nomogram was improved

**Fig. 7:**
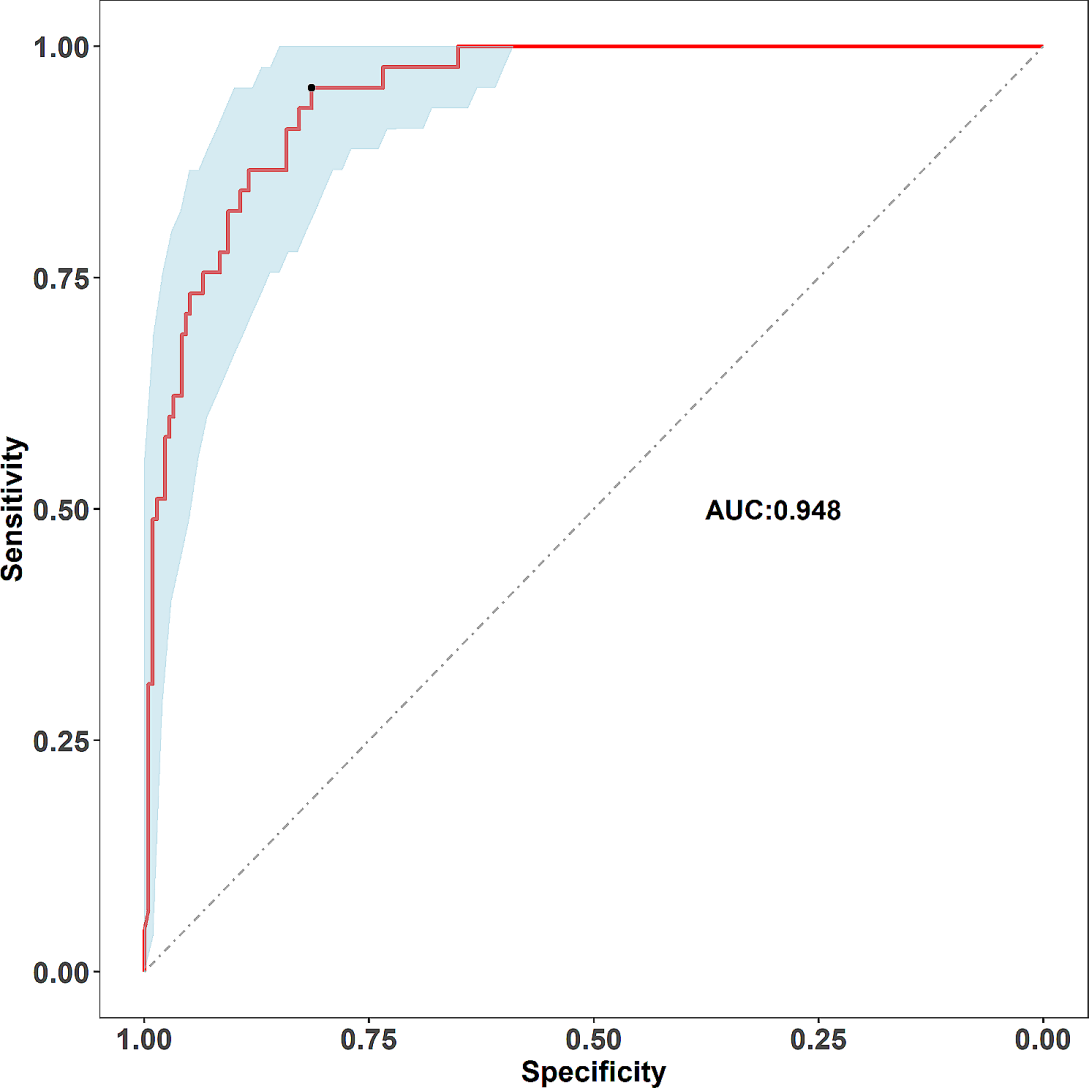
ROC curve generated to assess the performance of the SSBO nomogram. The area under the ROC curve for the prediction nomogram was 0.948 (95% confidence interval: 0.814–0.956). ROC, receiving operator characteristic

In this cohort, the calibration curve of the SSBO risk nomogram showed good agreement (Fig. 6). The area under the receiver operating characteristic curve was 0.948 (95% CI: 0.814–0.956). Bootstrapping with 500 replicates was performed for internal validation (Fig. 7).

### Clinical application

**Fig. 8:**
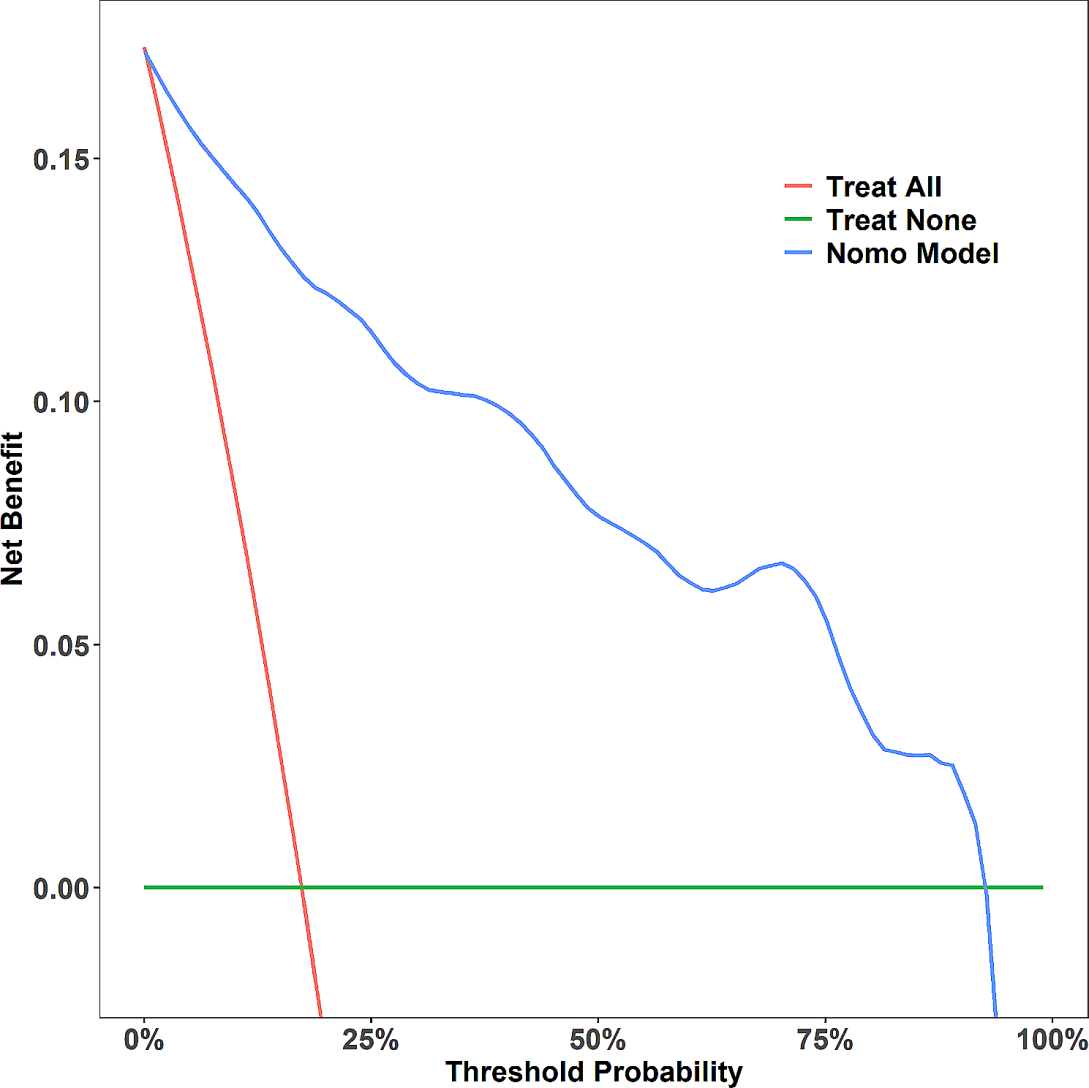
Decision curve analysis was performed to evaluate the prediction model. The blue line represents outcomes of the prediction model and the red line represents outcomes for all patients with SSBO. The green solid horizontal line indicates the absence of SSBO in any patients. The graph illustrates the expected net benefit per patient in relation to the nomogram prediction of SSBO risk. As the model curve extends, the net benefit increases. SSBO, severe small bowel obstruction

The DCA of the SSBO nomogram is presented in Fig. 8. The decision curve demonstrates that if the threshold probability of SSBO is greater than 10%, using this SSBO predictive nomogram provides additional benefits. This predictive model therefore has a high clinical applicability.

### Effects of the Houpu Paiqi mixture

Table 3 provides the baseline demographic information, clinical parameters, and laboratory results for patients treated with the Houpu Paiqi mixture and those who did not receive this treatment. Preoperative data did not differ significantly between the two groups. However, there were statistically significant differences in postoperative measures between the two groups, including postoperative exhaust time, postoperative defecation time, time to first postoperative liquid feed, and length of stay in hospital.

**Table 3:** Characteristics of patients treated with and without the Houpu Paiqi mixture

Characteristic	Overall *N* = 89	Houpu *N* = 28	Non-Houpe *N* = 61	*P*-value
Age	62.0(49.0;70.0)	65.0;(53.2;75.0)	60.0(49.0;69.0)	0.192
Gender				0.312
Female	36 (40.4%)	14 (50.0%)	22 (36.1%)	
Male	53 (59.6%)	14 (50.0%)	39 (63.9%)	
BMI	21.7(19.5;23.9)	20.5(17.8;23.9)	21.8(20.0;23.6)	0.231
Body temperature	36.3 (36.20;36.70)	36.3 (36.10;36.70)	36.4 (36.20;36.70)	0.200
Heart rate	82.0 (80.00;96.00)	81.0 (80.00;96.00)	82.0 (80.00;96.00)	0.762
Emergency surgery				0.374
No	89(93.3%)	25(89.3%)	58(95.1%)	
Yes	6(6.74%)	3(10.7%)	3(4.92%)	
Nutritional support				0.507
No	77(86.5%)	5(17.9%)	7(11.5%)	
Yes	12(13.5%)	23(82.1%)	54(88.5%)	
Presence of gas and liquid level in X-ray				0.543
No	15 (16.9%)	6 (21.4%)	9 (14.8%)	
Yes	74 (83.1%)	22 (78.6%)	52 (85.2%)	
Abdominal operations times				0.512
0	37 (41.6%)	9 (32.1%)	28 (45.9%)	
1	42 (47.2%)	15 (53.6%)	27 (44.3%)	
2	9 (10.1%)	4 (14.3%)	5 (8.20%)	
3	1 (1.12%)	0 (0%)	1 (1.6%)	
Chronic diseases				0.879
No	28 (31.5%)	8 (28.6%)	20 (32.8%)	
Yes	61 (68.5%)	20 (71.4%)	41 (67.2%)	
Length of stay	14.0 (11.00;19.00)	16.5 (11.75;20.00)	13.0 (11.00;17.00)	0.221
Postoperative exhaust time	4.0 (3.00;4.00)	3.0 (2.00;4.00)	4.0 (3.00;5.00)	0.004
Postoperative defecation time	5.0 (4.00;6.00)	4.0 (3.00;5.00)	6.0 (5.00;6.00)	0.001
The time of the first liquid diet	6.0 (6.00;8.00)	5.5 (4.00; 7.25)	7.0 (6.00;8.00)	0.004
Length of postoperative hospital stay	10.0 (8.00;14.00)	8.50 (6.00;13.00)	11.0 (9.00;14.00)	0.012
Drainage tube removal time	7.0 (6.00;9.00)	7.5 (7.00;9.25)	7.0 (6.00;9.00)	0.155
Time of gastric tube removal	3.0 (2.00;5.00)	3.0 (3.00;4.25)	3.0 (2.00;5.00)	0.462
White blood cell	6.88(5.29;10.6)	7.21(5.62;10.5)	6.68(5.18;10.6)	0.714
White blood cells after intervention	7.07(5.67;8.79)	6.76(5.16;8.32)	7.27(6.20;9.20)	0.267

Categorical data are expressed as percentages, and continuous data are expressed as medians (upper and lower quartiles)

## Discussion

In this study, we developed and internally validated an individualized prediction nomogram for SSBO. We identified eight easily assessed variables that could be used by medical practitioner to predict the occurrence of SSBO. These variables were: age, peritonitis, intestinal resection and anastomosis, complications, operation time, APACHE II score, WBC count, and procalcitonin levels. The nomogram demonstrated a good performance in SSBO risk prediction. The internal validation of the model revealed its strong discrimination and calibration abilities. Additionally, DCA demonstrated that this model has clinical significance in decision-making across a range of probability thresholds.

The overall function of the gut typically declines with age, potentially due to reduced exercise and changes in dietary habits [21]. Additionally, the intestinal mucosa of older individuals is more susceptible to inflammation than that of younger individuals, and this inflammatory response can exacerbate intestinal tissue damage [22–24]. Increased WBC counts and procalcitonin levels often indicate inflammation [25, 26]. The worsening of obstruction symptoms and emergence of signs of peritonitis prior to surgery suggests severe inflammation in the abdominal cavity; this can result in significantly impaired intestinal function and an extended postoperative recovery time. Consequently, older patients with preoperative signs of peritonitis are more likely to develop SSBO after surgery.

Operation time and the use of enterectomy and anastomosis were also independent risk factors for SSBO. The resection and anastomosis procedure not only alters the original anatomical structure of the small bowel but also disrupts intestinal continuity. Additionally, it causes traction stimulation of the normal small bowel, which slows peristalsis. Compared with adhesion-release surgery alone, enterectomy and anastomosis prolong the operation time, thus increasing the duration of anesthesia and delaying postoperative intestinal function recovery [27].

APACHE II is a non-specific critical disease scoring system commonly employed in clinical practice [13, 28]. This study identified the APACHE II score as an independent risk factor for SSBO. Postoperative complications included hypoproteinemia, sepsis, septic shock, type I respiratory failure, anastomotic fistulas, and massive ascites. These complications cause a deterioration in the general health of the patient. Physiological compensation diminishes, resulting in decreased stress tolerance, immunity, and overall ability to recover from surgical trauma, and increasing the likelihood of SSBO.

The Chinese medicine preparation Houpu Paiqi has been used in patients after adhesive intestinal obstruction surgery, achieving satisfactory results. In our study we superficially demonstrated that Houpu Paiqi mixture can promotes intestinal function recovery in clinical settings. Its main components are honokiol (Magnolia officinalis), naringin, and emodin [8–10]. Honokiol (HKL) is a bioactive compound extracted from several species of magnolia officinalis. HKL has a variety of biological activities, including anti-inflammatory, anti-platelet, anti-angiogenesis, anti-tumor, anti-oxidation, and anti-cardiac hypertrophy. Additionally, HKL can be considered a natural SIRT3 activator, as it can directly bind to SIRT3 and improve its enzyme activity [29–32]. Tianli Shen et al. has been demonstrated that HKL, as a candidate SIRT3 activator, may be a novel therapeutic agent for reducing ROS formation, inflammation, and NLRP3 activation, thereby improving postoperative PA formation. Naringin (NG) is a natural flavonoid (a flavanone glycoside) that has been shown to have anti-inflammatory, antioxidant, antiulcer, antiosteoporosis, and anticancer effects [33–35]. Shufeng Wang et al. reported that both ROS and MDA levels decreased in the adhesive tissues of the NG treatment groups, with more pronounced results in the high-dose NG group. This suggests that NG inhibited the degree of oxidative stress in adhesion tissues, and its mechanisms may be related to the ability of NG to ameliorate mitochondrial dysfunction. NG can be used as a drug to reduce the severity and incidence of adhesion, promote the early recovery of postoperative gastrointestinal function, and accelerate recovery after gastrointestinal surgery.

Emodin is a natural secondary plant product that was originally isolated from the rhizomes of Rheum *palmatum*. In traditional Chinese medicine, emodin is used as an anti-inflammatory agent and to improve visceral stasis and promote gut movement. Previous studies have demonstrated that emodin has various effects, including antiviral, antibacterial, antiallergenic, anti-osteoporotic, antidiabetic, anti-inflammatory, and antitumor effects, and it can reduce oxidative stress [36, 37]. Xuqi Li et al. demonstrated that emodin prevents adhesion formation in several ways, including the inhibition of inflammation, alleviation of oxidative stress, and promotion of intestinal tract movements. The main effect of emodin is to prevent abdominal adhesion formation by blocking the TGF-β signaling pathway. Their results presented in this study strongly suggest that emodin can be an effective drug for the prevention of postoperative adhesion formation.

In this study, patients treated with Houpu Paiqi mixture had shorter hospital stays than those not treated with Houpu Paiqi mixture (8.5 vs. 11 day). Furthermore, the postoperative exhaust time, the postoperative defecation time, and the time to first postoperative liquid feed were shorter in patients treated with Houpu Paiqi mixture than in those who did not receive this treatment. While there was no statistically significant difference in the WBC counts between the two groups, the WBC count in the Houpu Paiqi-treated group showed a downward trend, compared to an increased trend in patients not receiving this treatment. This suggests that the Houpu Paiqi mixture promotes intestinal function recovery, inhibits inflammation, and relieves oxidative stress. These findings are consistent with those of previous studies, which have also demonstrated a reduction in the formation of postoperative adhesions.

### Limitations

The present study has several limitations. First, the retrospective nature of the study makes it difficult to eliminate bias. Additionally, the sample size was relatively small, which affected the generalizability of the findings. Second, this was a single-center study. Consequently, these data may not accurately represent the entire patient population. Third, it is important to acknowledge that the data collection was incomplete, and the risk factor analysis did not encompass all the potential factors that could predict SSBO. To address these limitations, external validation of our findings and prospective multicenter studies with large patient cohorts are required.

## Conclusion

We developed a model to predict SSBO in patients undergoing surgery for small bowel obstruction, using factors that can be easily assessed in a clinical setting. In addition, previously complicated regression equations have been converted into user-friendly graphs, making it easier for clinicians to interpret and apply this model. In addition, our study superficially illustrates Houpu Paiqi mixture aids in the recovery of intestinal function postoperative. However, larger sample sizes and high-quality prospective studies are required to validate this model further.

## Data Availability

The data used to support the findings of this study are available from the corresponding author upon request.
